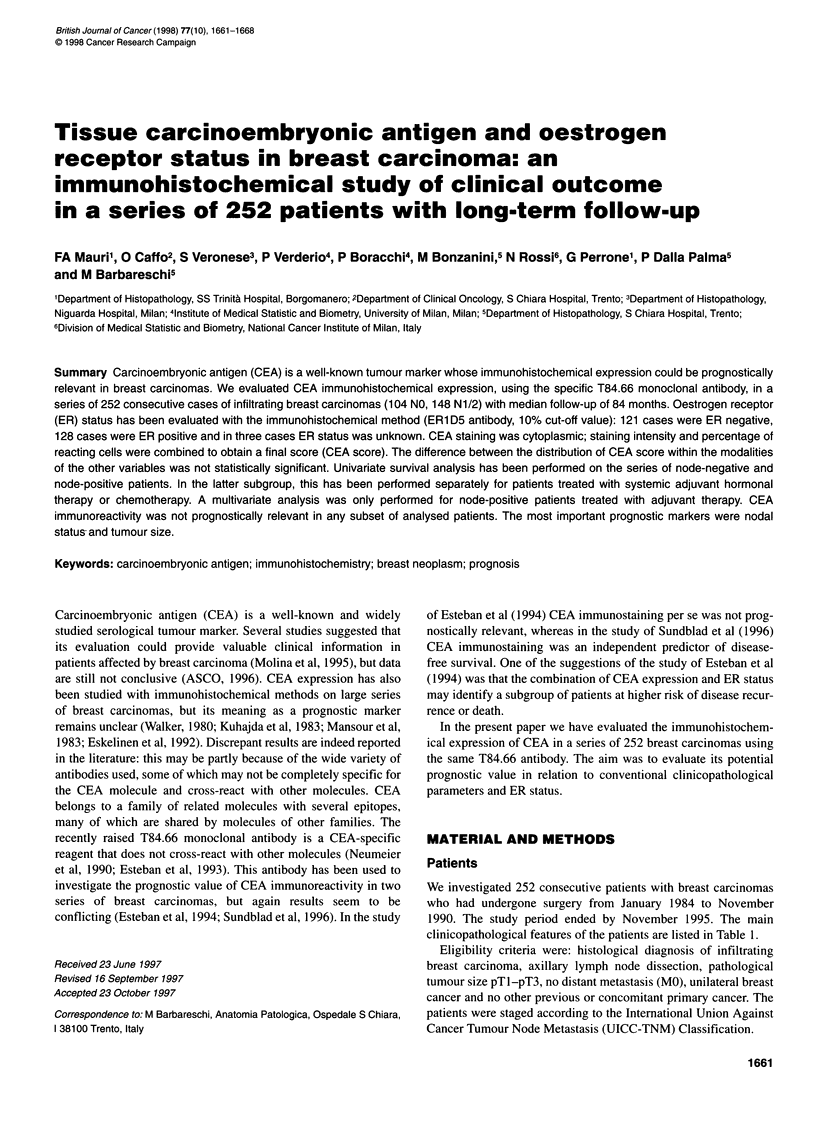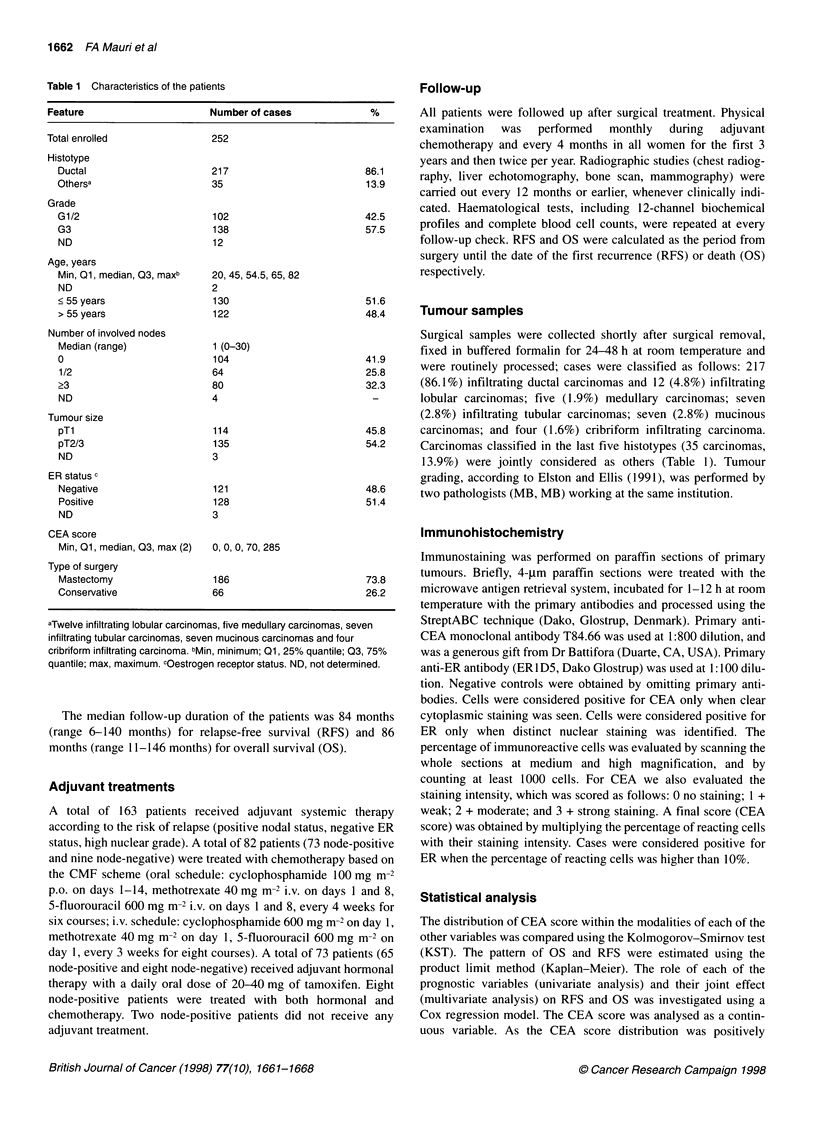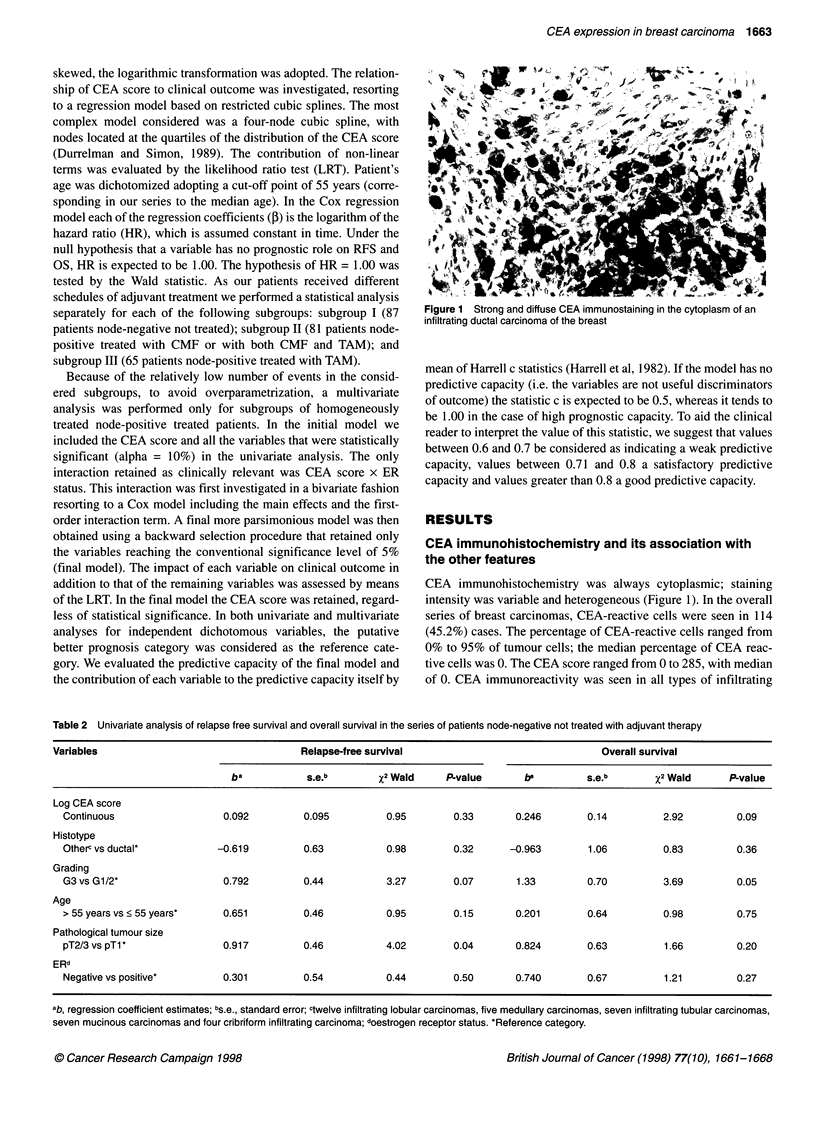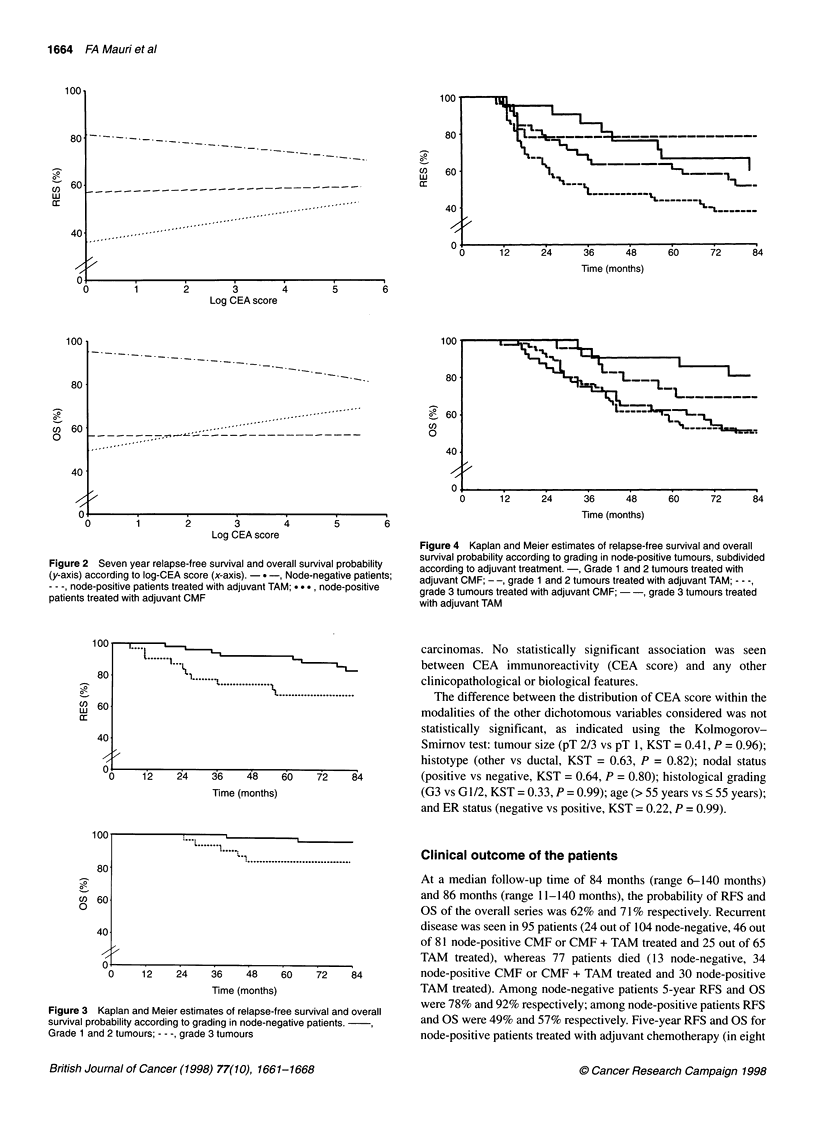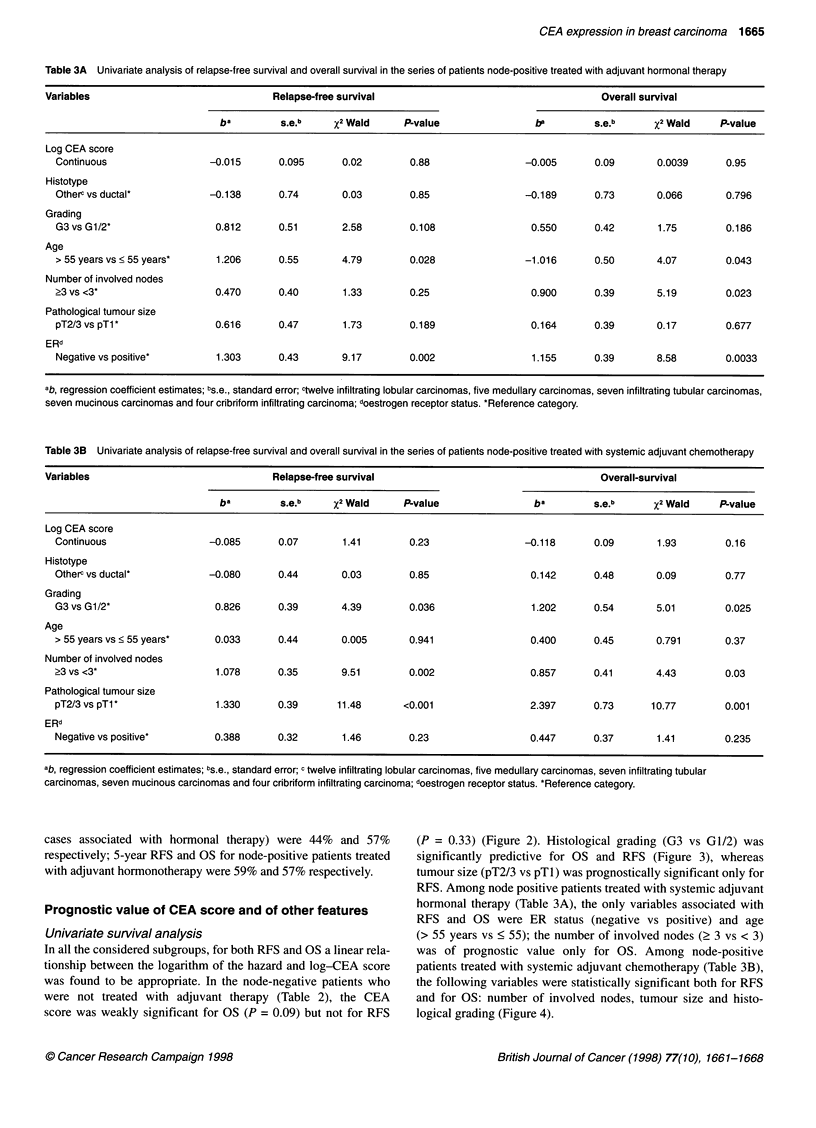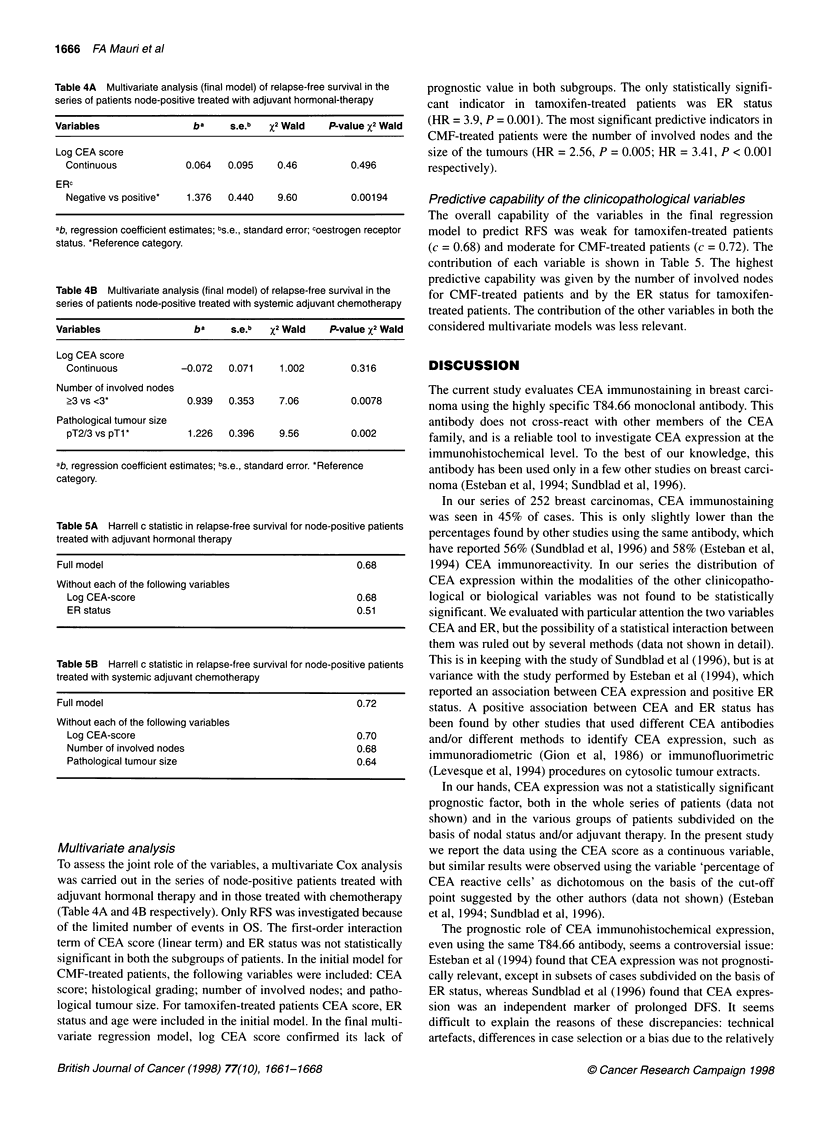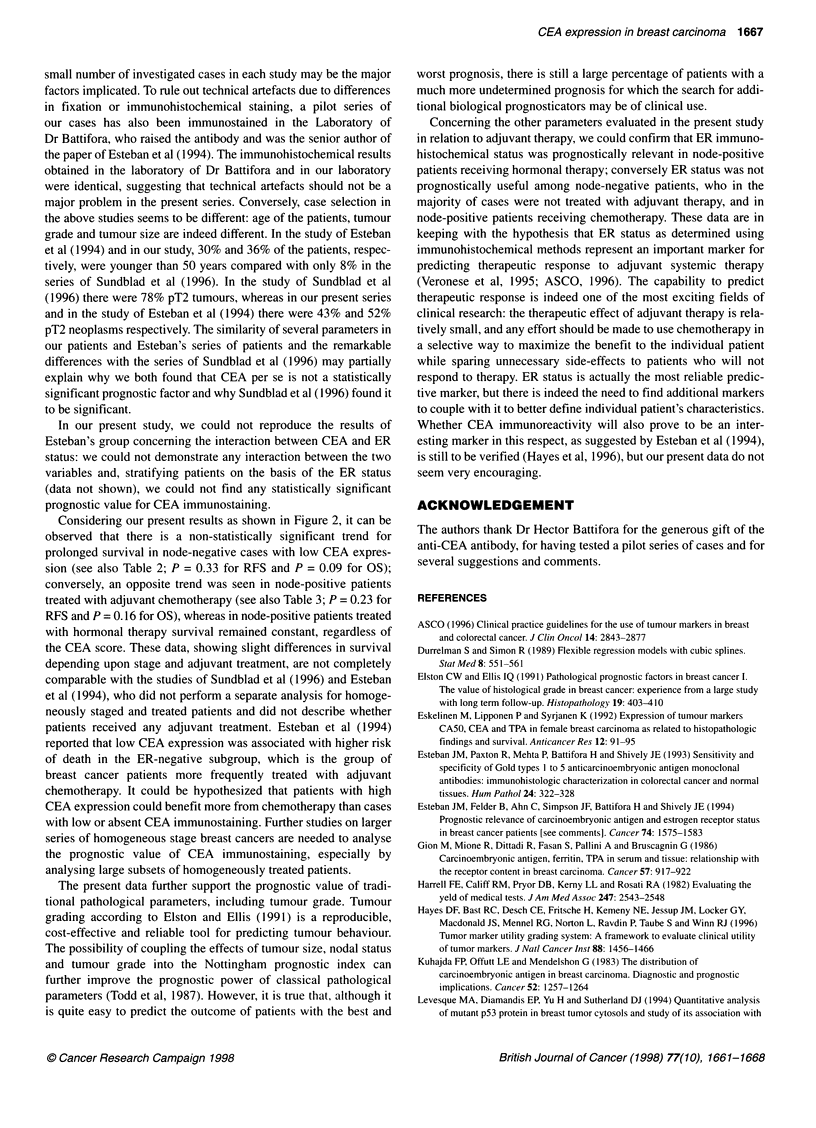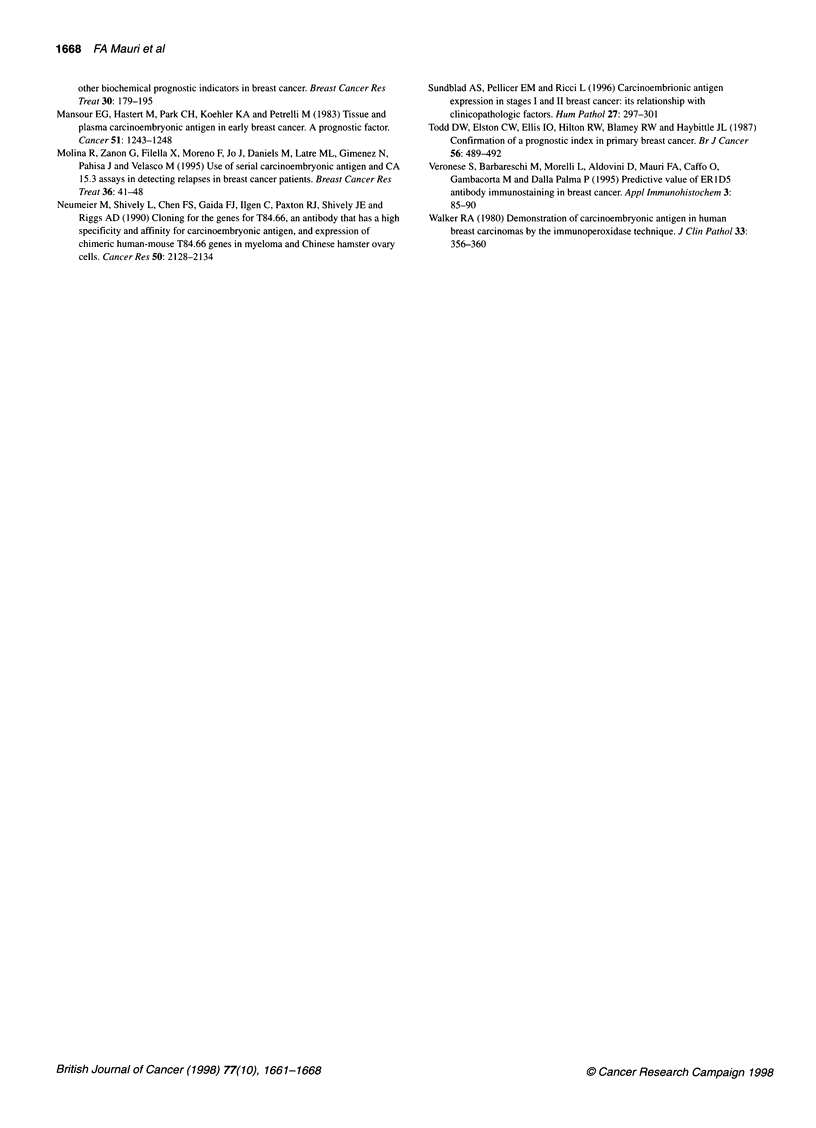# Tissue carcinoembryonic antigen and oestrogen receptor status in breast carcinoma: an immunohistochemical study of clinical outcome in a series of 252 patients with long-term follow-up.

**DOI:** 10.1038/bjc.1998.273

**Published:** 1998-05

**Authors:** F. A. Mauri, O. Caffo, S. Veronese, P. Verderio, P. Boracchi, M. Bonzanini, N. Rossi, G. Perrone, P. Dalla Palma, M. Barbareschi

**Affiliations:** Department of Histopathology, SS TrinitÃ Hospital, Borgomanero, Italy.

## Abstract

**Images:**


British Journal of Cancer (1998) 77(10), 1661–1668
© 1998 Cancer Research Campaign

### Tissue carcinoembryonic antigen and oestrogen receptor status in breast carcinoma: an immunohistochemical study of clinical outcome in a series of 252 patients with long-term follow-up

**FA Mauri[1], O Caffo[2], S Veronese[3], P Verderio[4], P Boracchi[4], M Bonzanini,[5] N Rossi[6], G Perrone[1], P Dalla Palma[5] and M Barbareschi[5]**

[1]Department of Histopathology, SS Trinità Hospital, Borgomanero; [2]Department of Clinical Oncology, S Chiara Hospital, Trento; [3]Department of Histopathology, Niguarda Hospital, Milan; [4]Institute of Medical Statistic and Biometry, University of Milan, Milan; [5]Department of Histopathology, S Chiara Hospital, Trento; [6]Division of Medical Statistic and Biometry, National Cancer Institute of Milan, Italy

**Summary** Carcinoembryonic antigen (CEA) is a well-known tumour marker whose immunohistochemical expression could be prognostically relevant in breast carcinomas. We evaluated CEA immunohistochemical expression, using the specific T84.66 monoclonal antibody, in a series of 252 consecutive cases of infiltrating breast carcinomas (104 N0, 148 N1/2) with median follow-up of 84 months. Oestrogen receptor (ER) status has been evaluated with the immunohistochemical method (ER1D5 antibody, 10% cut-off value): 121 cases were ER negative, 128 cases were ER positive and in three cases ER status was unknown. CEA staining was cytoplasmic; staining intensity and percentage of reacting cells were combined to obtain a final score (CEA score). The difference between the distribution of CEA score within the modalities of the other variables was not statistically significant. Univariate survival analysis has been performed on the series of node-negative and node-positive patients. In the latter subgroup, this has been performed separately for patients treated with systemic adjuvant hormonal therapy or chemotherapy. A multivariate analysis was only performed for node-positive patients treated with adjuvant therapy. CEA immunoreactivity was not prognostically relevant in any subset of analysed patients. The most important prognostic markers were nodal status and tumour size.

**Keywords:** carcinoembryonic antigen; immunohistochemistry; breast neoplasm; prognosis

Carcinoembryonic antigen (CEA) is a well-known and widely studied serological tumour marker. Several studies suggested that its evaluation could provide valuable clinical information in patients affected by breast carcinoma (Molina et al, 1995), but data are still not conclusive (ASCO, 1996). CEA expression has also been studied with immunohistochemical methods on large series of breast carcinomas, but its meaning as a prognostic marker remains unclear (Walker, 1980; Kuhajda et al, 1983; Mansour et al, 1983; Eskelinen et al, 1992). Discrepant results are indeed reported in the literature: this may be partly because of the wide variety of antibodies used, some of which may not be completely specific for the CEA molecule and cross-react with other molecules. CEA belongs to a family of related molecules with several epitopes, many of which are shared by molecules of other families. The recently raised T84.66 monoclonal antibody is a CEA-specific reagent that does not cross-react with other molecules (Neumeier et al, 1990; Esteban et al, 1993). This antibody has been used to investigate the prognostic value of CEA immunoreactivity in two series of breast carcinomas, but again results seem to be conflicting (Esteban et al, 1994; Sundblad et al, 1996). In the study of Esteban et al (1994) CEA immunostaining per se was not prognostically relevant, whereas in the study of Sundblad et al (1996) CEA immunostaining was an independent predictor of disease-free survival. One of the suggestions of the study of Esteban et al (1994) was that the combination of CEA expression and ER status may identify a subgroup of patients at higher risk of disease recurrence or death.

In the present paper we have evaluated the immunohistochemical expression of CEA in a series of 252 breast carcinomas using the same T84.66 antibody. The aim was to evaluate its potential prognostic value in relation to conventional clinicopathological parameters and ER status.

*Received 23 June 1997*
*Revised 16 September 1997*
*Accepted 23 October 1997*

*Correspondence to:* M Barbareschi, Anatomia Patologica, Ospedale S Chiara, I 38100 Trento, Italy

### MATERIAL AND METHODS

### Patients

We investigated 252 consecutive patients with breast carcinomas who had undergone surgery from January 1984 to November 1990. The study period ended by November 1995. The main clinicopathological features of the patients are listed in Table 1.

Eligibility criteria were: histological diagnosis of infiltrating breast carcinoma, axillary lymph node dissection, pathological tumour size pT1–pT3, no distant metastasis (M0), unilateral breast cancer and no other previous or concomitant primary cancer. The patients were staged according to the International Union Against Cancer Tumour Node Metastasis (UICC-TNM) Classification.

**Table 1** Characteristics of the patients

| Feature | Number of cases | % |
|---|---|---|
| Total enrolled | 252 | |
| Histotype | | |
| Ductal | 217 | 86.1 |
| Others[a] | 35 | 13.9 |
| Grade | | |
| G1/2 | 102 | 42.5 |
| G3 | 138 | 57.5 |
| ND | 12 | |
| Age, years | | |
| Min, Q1, median, Q3, max[b] | 20, 45, 54.5, 65, 82 | |
| ND | 2 | |
| ≤ 55 years | 130 | 51.6 |
| > 55 years | 122 | 48.4 |
| Number of involved nodes | | |
| Median (range) | 1 (0–30) | |
| 0 | 104 | 41.9 |
| 1/2 | 64 | 25.8 |
| ≥3 | 80 | 32.3 |
| ND | 4 | – |
| Tumour size | | |
| pT1 | 114 | 45.8 |
| pT2/3 | 135 | 54.2 |
| ND | 3 | |
| ER status [c] | | |
| Negative | 121 | 48.6 |
| Positive | 128 | 51.4 |
| ND | 3 | |
| CEA score | | |
| Min, Q1, median, Q3, max (2) | 0, 0, 0, 70, 285 | |
| Type of surgery | | |
| Mastectomy | 186 | 73.8 |
| Conservative | 66 | 26.2 |

[a]Twelve infiltrating lobular carcinomas, five medullary carcinomas, seven infiltrating tubular carcinomas, seven mucinous carcinomas and four cribriform infiltrating carcinoma. [b]Min, minimum; Q1, 25% quantile; Q3, 75% quantile; max, maximum. [c]Oestrogen receptor status. ND, not determined.

The median follow-up duration of the patients was 84 months (range 6–140 months) for relapse-free survival (RFS) and 86 months (range 11–146 months) for overall survival (OS).

### Adjuvant treatments

A total of 163 patients received adjuvant systemic therapy according to the risk of relapse (positive nodal status, negative ER status, high nuclear grade). A total of 82 patients (73 node-positive and nine node-negative) were treated with chemotherapy based on the CMF scheme (oral schedule: cyclophosphamide 100 mg $m^{-2}$ p.o. on days 1–14, methotrexate 40 mg $m^{-2}$ i.v. on days 1 and 8, 5-fluorouracil 600 mg $m^{-2}$ i.v. on days 1 and 8, every 4 weeks for six courses; i.v. schedule: cyclophosphamide 600 mg $m^{-2}$ on day 1, methotrexate 40 mg $m^{-2}$ on day 1, 5-fluorouracil 600 mg $m^{-2}$ on day 1, every 3 weeks for eight courses). A total of 73 patients (65 node-positive and eight node-negative) received adjuvant hormonal therapy with a daily oral dose of 20–40 mg of tamoxifen. Eight node-positive patients were treated with both hormonal and chemotherapy. Two node-positive patients did not receive any adjuvant treatment.

### Follow-up

All patients were followed up after surgical treatment. Physical examination was performed monthly during adjuvant chemotherapy and every 4 months in all women for the first 3 years and then twice per year. Radiographic studies (chest radiography, liver echotomography, bone scan, mammography) were carried out every 12 months or earlier, whenever clinically indicated. Haematological tests, including 12-channel biochemical profiles and complete blood cell counts, were repeated at every follow-up check. RFS and OS were calculated as the period from surgery until the date of the first recurrence (RFS) or death (OS) respectively.

### Tumour samples

Surgical samples were collected shortly after surgical removal, fixed in buffered formalin for 24–48 h at room temperature and were routinely processed; cases were classified as follows: 217 (86.1%) infiltrating ductal carcinomas and 12 (4.8%) infiltrating lobular carcinomas; five (1.9%) medullary carcinomas; seven (2.8%) infiltrating tubular carcinomas; seven (2.8%) mucinous carcinomas; and four (1.6%) cribriform infiltrating carcinoma. Carcinomas classified in the last five histotypes (35 carcinomas, 13.9%) were jointly considered as others (Table 1). Tumour grading, according to Elston and Ellis (1991), was performed by two pathologists (MB, MB) working at the same institution.

### Immunohistochemistry

Immunostaining was performed on paraffin sections of primary tumours. Briefly, 4-μm paraffin sections were treated with the microwave antigen retrieval system, incubated for 1–12 h at room temperature with the primary antibodies and processed using the StreptABC technique (Dako, Glostrup, Denmark). Primary anti-CEA monoclonal antibody T84.66 was used at 1:800 dilution, and was a generous gift from Dr Battifora (Duarte, CA, USA). Primary anti-ER antibody (ER1D5, Dako Glostrup) was used at 1:100 dilution. Negative controls were obtained by omitting primary antibodies. Cells were considered positive for CEA only when clear cytoplasmic staining was seen. Cells were considered positive for ER only when distinct nuclear staining was identified. The percentage of immunoreactive cells was evaluated by scanning the whole sections at medium and high magnification, and by counting at least 1000 cells. For CEA we also evaluated the staining intensity, which was scored as follows: 0 no staining; 1 + weak; 2 + moderate; and 3 + strong staining. A final score (CEA score) was obtained by multiplying the percentage of reacting cells with their staining intensity. Cases were considered positive for ER when the percentage of reacting cells was higher than 10%.

### Statistical analysis

The distribution of CEA score within the modalities of each of the other variables was compared using the Kolmogorov–Smirnov test (KST). The pattern of OS and RFS were estimated using the product limit method (Kaplan–Meier). The role of each of the prognostic variables (univariate analysis) and their joint effect (multivariate analysis) on RFS and OS was investigated using a Cox regression model. The CEA score was analysed as a continuous variable. As the CEA score distribution was positively

© Cancer Research Campaign 1998

skewed, the logarithmic transformation was adopted. The relationship of CEA score to clinical outcome was investigated, resorting to a regression model based on restricted cubic splines. The most complex model considered was a four-node cubic spline, with nodes located at the quartiles of the distribution of the CEA score (Durrelman and Simon, 1989). The contribution of non-linear terms was evaluated by the likelihood ratio test (LRT). Patient's age was dichotomized adopting a cut-off point of 55 years (corresponding in our series to the median age). In the Cox regression model each of the regression coefficients (β) is the logarithm of the hazard ratio (HR), which is assumed constant in time. Under the null hypothesis that a variable has no prognostic role on RFS and OS, HR is expected to be 1.00. The hypothesis of HR = 1.00 was tested by the Wald statistic. As our patients received different schedules of adjuvant treatment we performed a statistical analysis separately for each of the following subgroups: subgroup I (87 patients node-negative not treated); subgroup II (81 patients node-positive treated with CMF or with both CMF and TAM); and subgroup III (65 patients node-positive treated with TAM).

Because of the relatively low number of events in the considered subgroups, to avoid overparametrization, a multivariate analysis was performed only for subgroups of homogeneously treated node-positive treated patients. In the initial model we included the CEA score and all the variables that were statistically significant (alpha = 10%) in the univariate analysis. The only interaction retained as clinically relevant was CEA score × ER status. This interaction was first investigated in a bivariate fashion resorting to a Cox model including the main effects and the first-order interaction term. A final more parsimonious model was then obtained using a backward selection procedure that retained only the variables reaching the conventional significance level of 5% (final model). The impact of each variable on clinical outcome in addition to that of the remaining variables was assessed by means of the LRT. In the final model the CEA score was retained, regardless of statistical significance. In both univariate and multivariate analyses for independent dichotomous variables, the putative better prognosis category was considered as the reference category. We evaluated the predictive capacity of the final model and the contribution of each variable to the predictive capacity itself by

**Figure 1** Strong and diffuse CEA immunostaining in the cytoplasm of an infiltrating ductal carcinoma of the breast mean of Harrell c statistics (Harrell et al, 1982). If the model has no predictive capacity (i.e. the variables are not useful discriminators of outcome) the statistic c is expected to be 0.5, whereas it tends to be 1.00 in the case of high prognostic capacity. To aid the clinical reader to interpret the value of this statistic, we suggest that values between 0.6 and 0.7 be considered as indicating a weak predictive capacity, values between 0.71 and 0.8 a satisfactory predictive capacity and values greater than 0.8 a good predictive capacity.

### RESULTS

### CEA immunohistochemistry and its association with the other features

CEA immunohistochemistry was always cytoplasmic; staining intensity was variable and heterogeneous (Figure 1). In the overall series of breast carcinomas, CEA-reactive cells were seen in 114 (45.2%) cases. The percentage of CEA-reactive cells ranged from 0% to 95% of tumour cells; the median percentage of CEA reactive cells was 0. The CEA score ranged from 0 to 285, with median of 0. CEA immunoreactivity was seen in all types of infiltrating

**Table 2** Univariate analysis of relapse free survival and overall survival in the series of patients node-negative not treated with adjuvant therapy

| Variables | Relapse-free survival | | | | Overall survival | | | |
|---|---|---|---|---|---|---|---|---|
| | *b*[a] | s.e.[b] | $\chi^2$ Wald | *P*-value | *b*[a] | s.e.[b] | $\chi^2$ Wald | *P*-value |
| Log CEA score | | | | | | | | |
| Continuous | 0.092 | 0.095 | 0.95 | 0.33 | 0.246 | 0.14 | 2.92 | 0.09 |
| Histotype | | | | | | | | |
| Other[c] vs ductal* | –0.619 | 0.63 | 0.98 | 0.32 | –0.963 | 1.06 | 0.83 | 0.36 |
| Grading | | | | | | | | |
| G3 vs G1/2* | 0.792 | 0.44 | 3.27 | 0.07 | 1.33 | 0.70 | 3.69 | 0.05 |
| Age | | | | | | | | |
| > 55 years vs ≤ 55 years* | 0.651 | 0.46 | 0.95 | 0.15 | 0.201 | 0.64 | 0.98 | 0.75 |
| Pathological tumour size | | | | | | | | |
| pT2/3 vs pT1* | 0.917 | 0.46 | 4.02 | 0.04 | 0.824 | 0.63 | 1.66 | 0.20 |
| ER[d] | | | | | | | | |
| Negative vs positive* | 0.301 | 0.54 | 0.44 | 0.50 | 0.740 | 0.67 | 1.21 | 0.27 |

[a]*b*, regression coefficient estimates; [b]s.e., standard error; [c]twelve infiltrating lobular carcinomas, five medullary carcinomas, seven infiltrating tubular carcinomas, seven mucinous carcinomas and four cribriform infiltrating carcinoma; [d]oestrogen receptor status. *Reference category.

© Cancer Research Campaign 1998 

**Figure 2** Seven year relapse-free survival and overall survival probability (*y*-axis) according to log-CEA score (*x*-axis). — • —, Node-negative patients; - - -, node-positive patients treated with adjuvant TAM; • • •, node-positive patients treated with adjuvant CMF

**Figure 4** Kaplan and Meier estimates of relapse-free survival and overall survival probability according to grading in node-positive tumours, subdivided according to adjuvant treatment. —, Grade 1 and 2 tumours treated with adjuvant CMF; – –, grade 1 and 2 tumours treated with adjuvant TAM; - - -, grade 3 tumours treated with adjuvant CMF; — —, grade 3 tumours treated with adjuvant TAM

**Figure 3** Kaplan and Meier estimates of relapse-free survival and overall survival probability according to grading in node-negative patients. ——, Grade 1 and 2 tumours; - - -, grade 3 tumours carcinomas. No statistically significant association was seen between CEA immunoreactivity (CEA score) and any other clinicopathological or biological features.

The difference between the distribution of CEA score within the modalities of the other dichotomous variables considered was not statistically significant, as indicated using the Kolmogorov–Smirnov test: tumour size (pT 2/3 vs pT 1, KST = 0.41, $P = 0.96$); histotype (other vs ductal, KST = 0.63, $P = 0.82$); nodal status (positive vs negative, KST = 0.64, $P = 0.80$); histological grading (G3 vs G1/2, KST = 0.33, $P = 0.99$); age (> 55 years vs ≤ 55 years); and ER status (negative vs positive, KST = 0.22, $P = 0.99$).

### Clinical outcome of the patients

At a median follow-up time of 84 months (range 6–140 months) and 86 months (range 11–140 months), the probability of RFS and OS of the overall series was 62% and 71% respectively. Recurrent disease was seen in 95 patients (24 out of 104 node-negative, 46 out of 81 node-positive CMF or CMF + TAM treated and 25 out of 65 TAM treated), whereas 77 patients died (13 node-negative, 34 node-positive CMF or CMF + TAM treated and 30 node-positive TAM treated). Among node-negative patients 5-year RFS and OS were 78% and 92% respectively; among node-positive patients RFS and OS were 49% and 57% respectively. Five-year RFS and OS for node-positive patients treated with adjuvant chemotherapy (in eight

© Cancer Research Campaign 1998

**Table 3A** Univariate analysis of relapse-free survival and overall survival in the series of patients node-positive treated with adjuvant hormonal therapy

| Variables | Relapse-free survival | | | | Overall survival | | | |
|---|---|---|---|---|---|---|---|---|
| | $b^a$ | s.e.[b] | $\chi^2$ Wald | *P*-value | $b^a$ | s.e.[b] | $\chi^2$ Wald | *P*-value |
| Log CEA score | | | | | | | | |
| Continuous | −0.015 | 0.095 | 0.02 | 0.88 | −0.005 | 0.09 | 0.0039 | 0.95 |
| Histotype | | | | | | | | |
| Other[c] vs ductal* | −0.138 | 0.74 | 0.03 | 0.85 | −0.189 | 0.73 | 0.066 | 0.796 |
| Grading | | | | | | | | |
| G3 vs G1/2* | 0.812 | 0.51 | 2.58 | 0.108 | 0.550 | 0.42 | 1.75 | 0.186 |
| Age | | | | | | | | |
| > 55 years vs ≤ 55 years* | 1.206 | 0.55 | 4.79 | 0.028 | −1.016 | 0.50 | 4.07 | 0.043 |
| Number of involved nodes | | | | | | | | |
| ≥3 vs <3* | 0.470 | 0.40 | 1.33 | 0.25 | 0.900 | 0.39 | 5.19 | 0.023 |
| Pathological tumour size | | | | | | | | |
| pT2/3 vs pT1* | 0.616 | 0.47 | 1.73 | 0.189 | 0.164 | 0.39 | 0.17 | 0.677 |
| ER[d] | | | | | | | | |
| Negative vs positive* | 1.303 | 0.43 | 9.17 | 0.002 | 1.155 | 0.39 | 8.58 | 0.0033 |

[a]*b*, regression coefficient estimates; [b]s.e., standard error; [c]twelve infiltrating lobular carcinomas, five medullary carcinomas, seven infiltrating tubular carcinomas, seven mucinous carcinomas and four cribriform infiltrating carcinoma; [d]oestrogen receptor status. *Reference category.

**Table 3B** Univariate analysis of relapse-free survival and overall survival in the series of patients node-positive treated with systemic adjuvant chemotherapy

| Variables | Relapse-free survival | | | | Overall-survival | | | |
|---|---|---|---|---|---|---|---|---|
| | $b^a$ | s.e.[b] | $\chi^2$ Wald | *P*-value | $b^a$ | s.e.[b] | $\chi^2$ Wald | *P*-value |
| Log CEA score | | | | | | | | |
| Continuous | −0.085 | 0.07 | 1.41 | 0.23 | −0.118 | 0.09 | 1.93 | 0.16 |
| Histotype | | | | | | | | |
| Other[c] vs ductal* | −0.080 | 0.44 | 0.03 | 0.85 | 0.142 | 0.48 | 0.09 | 0.77 |
| Grading | | | | | | | | |
| G3 vs G1/2* | 0.826 | 0.39 | 4.39 | 0.036 | 1.202 | 0.54 | 5.01 | 0.025 |
| Age | | | | | | | | |
| > 55 years vs ≤ 55 years* | 0.033 | 0.44 | 0.005 | 0.941 | 0.400 | 0.45 | 0.791 | 0.37 |
| Number of involved nodes | | | | | | | | |
| ≥3 vs <3* | 1.078 | 0.35 | 9.51 | 0.002 | 0.857 | 0.41 | 4.43 | 0.03 |
| Pathological tumour size | | | | | | | | |
| pT2/3 vs pT1* | 1.330 | 0.39 | 11.48 | <0.001 | 2.397 | 0.73 | 10.77 | 0.001 |
| ER[d] | | | | | | | | |
| Negative vs positive* | 0.388 | 0.32 | 1.46 | 0.23 | 0.447 | 0.37 | 1.41 | 0.235 |

[a]*b*, regression coefficient estimates; [b]s.e., standard error; [c] twelve infiltrating lobular carcinomas, five medullary carcinomas, seven infiltrating tubular carcinomas, seven mucinous carcinomas and four cribriform infiltrating carcinoma; [d]oestrogen receptor status. *Reference category.

cases associated with hormonal therapy) were 44% and 57% respectively; 5-year RFS and OS for node-positive patients treated with adjuvant hormonotherapy were 59% and 57% respectively.

### Prognostic value of CEA score and of other features

### *Univariate survival analysis*

In all the considered subgroups, for both RFS and OS a linear relationship between the logarithm of the hazard and log–CEA score was found to be appropriate. In the node-negative patients who were not treated with adjuvant therapy (Table 2), the CEA score was weakly significant for OS ($P = 0.09$) but not for RFS ($P = 0.33$) (Figure 2). Histological grading (G3 vs G1/2) was significantly predictive for OS and RFS (Figure 3), whereas tumour size (pT2/3 vs pT1) was prognostically significant only for RFS. Among node positive patients treated with systemic adjuvant hormonal therapy (Table 3A), the only variables associated with RFS and OS were ER status (negative vs positive) and age (> 55 years vs ≤ 55); the number of involved nodes (≥ 3 vs < 3) was of prognostic value only for OS. Among node-positive patients treated with systemic adjuvant chemotherapy (Table 3B), the following variables were statistically significant both for RFS and for OS: number of involved nodes, tumour size and histological grading (Figure 4).

**Table 4A** Multivariate analysis (final model) of relapse-free survival in the series of patients node-positive treated with adjuvant hormonal-therapy

| Variables | $b^a$ | s.e.[b] | $\chi^2$ Wald | P-value $\chi^2$ Wald |
|---|---|---|---|---|
| Log CEA score | | | | |
| Continuous | 0.064 | 0.095 | 0.46 | 0.496 |
| ER[c] | | | | |
| Negative vs positive* | 1.376 | 0.440 | 9.60 | 0.00194 |

[a]*b*, regression coefficient estimates; [b]s.e., standard error; [c]oestrogen receptor status. *Reference category.

**Table 4B** Multivariate analysis (final model) of relapse-free survival in the series of patients node-positive treated with systemic adjuvant chemotherapy

| Variables | $b^a$ | s.e.[b] | $\chi^2$ Wald | P-value $\chi^2$ Wald |
|---|---|---|---|---|
| Log CEA score | | | | |
| Continuous | –0.072 | 0.071 | 1.002 | 0.316 |
| Number of involved nodes | | | | |
| ≥3 vs <3* | 0.939 | 0.353 | 7.06 | 0.0078 |
| Pathological tumour size | | | | |
| pT2/3 vs pT1* | 1.226 | 0.396 | 9.56 | 0.002 |

[a]*b*, regression coefficient estimates; [b]s.e., standard error. *Reference category.

**Table 5A** Harrell c statistic in relapse-free survival for node-positive patients treated with adjuvant hormonal therapy

| | |
|---|---|
| Full model | 0.68 |
| Without each of the following variables | |
| Log CEA-score | 0.68 |
| ER status | 0.51 |

**Table 5B** Harrell c statistic in relapse-free survival for node-positive patients treated with systemic adjuvant chemotherapy

| | |
|---|---|
| Full model | 0.72 |
| Without each of the following variables | |
| Log CEA-score | 0.70 |
| Number of involved nodes | 0.68 |
| Pathological tumour size | 0.64 |

### *Multivariate analysis*

To assess the joint role of the variables, a multivariate Cox analysis was carried out in the series of node-positive patients treated with adjuvant hormonal therapy and in those treated with chemotherapy (Table 4A and 4B respectively). Only RFS was investigated because of the limited number of events in OS. The first-order interaction term of CEA score (linear term) and ER status was not statistically significant in both the subgroups of patients. In the initial model for CMF-treated patients, the following variables were included: CEA score; histological grading; number of involved nodes; and pathological tumour size. For tamoxifen-treated patients CEA score, ER status and age were included in the initial model. In the final multivariate regression model, log CEA score confirmed its lack of prognostic value in both subgroups. The only statistically significant indicator in tamoxifen-treated patients was ER status (HR = 3.9, $P = 0.001$). The most significant predictive indicators in CMF-treated patients were the number of involved nodes and the size of the tumours (HR = 2.56, $P = 0.005$; HR = 3.41, $P < 0.001$ respectively).

### *Predictive capability of the clinicopathological variables*

The overall capability of the variables in the final regression model to predict RFS was weak for tamoxifen-treated patients ($c = 0.68$) and moderate for CMF-treated patients ($c = 0.72$). The contribution of each variable is shown in Table 5. The highest predictive capability was given by the number of involved nodes for CMF-treated patients and by the ER status for tamoxifen-treated patients. The contribution of the other variables in both the considered multivariate models was less relevant.

### DISCUSSION

The current study evaluates CEA immunostaining in breast carcinoma using the highly specific T84.66 monoclonal antibody. This antibody does not cross-react with other members of the CEA family, and is a reliable tool to investigate CEA expression at the immunohistochemical level. To the best of our knowledge, this antibody has been used only in a few other studies on breast carcinoma (Esteban et al, 1994; Sundblad et al, 1996).

In our series of 252 breast carcinomas, CEA immunostaining was seen in 45% of cases. This is only slightly lower than the percentages found by other studies using the same antibody, which have reported 56% (Sundblad et al, 1996) and 58% (Esteban et al, 1994) CEA immunoreactivity. In our series the distribution of CEA expression within the modalities of the other clinicopathological or biological variables was not found to be statistically significant. We evaluated with particular attention the two variables CEA and ER, but the possibility of a statistical interaction between them was ruled out by several methods (data not shown in detail). This is in keeping with the study of Sundblad et al (1996), but is at variance with the study performed by Esteban et al (1994), which reported an association between CEA expression and positive ER status. A positive association between CEA and ER status has been found by other studies that used different CEA antibodies and/or different methods to identify CEA expression, such as immunoradiometric (Gion et al, 1986) or immunofluorimetric (Levesque et al, 1994) procedures on cytosolic tumour extracts.

In our hands, CEA expression was not a statistically significant prognostic factor, both in the whole series of patients (data not shown) and in the various groups of patients subdivided on the basis of nodal status and/or adjuvant therapy. In the present study we report the data using the CEA score as a continuous variable, but similar results were observed using the variable 'percentage of CEA reactive cells' as dichotomous on the basis of the cut-off point suggested by the other authors (data not shown) (Esteban et al, 1994; Sundblad et al, 1996).

The prognostic role of CEA immunohistochemical expression, even using the same T84.66 antibody, seems a controversial issue: Esteban et al (1994) found that CEA expression was not prognostically relevant, except in subsets of cases subdivided on the basis of ER status, whereas Sundblad et al (1996) found that CEA expression was an independent marker of prolonged DFS. It seems difficult to explain the reasons of these discrepancies: technical artefacts, differences in case selection or a bias due to the relatively

 © Cancer Research Campaign 1998

small number of investigated cases in each study may be the major factors implicated. To rule out technical artefacts due to differences in fixation or immunohistochemical staining, a pilot series of our cases has also been immunostained in the Laboratory of Dr Battifora, who raised the antibody and was the senior author of the paper of Esteban et al (1994). The immunohistochemical results obtained in the laboratory of Dr Battifora and in our laboratory were identical, suggesting that technical artefacts should not be a major problem in the present series. Conversely, case selection in the above studies seems to be different: age of the patients, tumour grade and tumour size are indeed different. In the study of Esteban et al (1994) and in our study, 30% and 36% of the patients, respectively, were younger than 50 years compared with only 8% in the series of Sundblad et al (1996). In the study of Sundblad et al (1996) there were 78% pT2 tumours, whereas in our present series and in the study of Esteban et al (1994) there were 43% and 52% pT2 neoplasms respectively. The similarity of several parameters in our patients and Esteban's series of patients and the remarkable differences with the series of Sundblad et al (1996) may partially explain why we both found that CEA per se is not a statistically significant prognostic factor and why Sundblad et al (1996) found it to be significant.

In our present study, we could not reproduce the results of Esteban's group concerning the interaction between CEA and ER status: we could not demonstrate any interaction between the two variables and, stratifying patients on the basis of the ER status (data not shown), we could not find any statistically significant prognostic value for CEA immunostaining.

Considering our present results as shown in Figure 2, it can be observed that there is a non-statistically significant trend for prolonged survival in node-negative cases with low CEA expression (see also Table 2; $P = 0.33$ for RFS and $P = 0.09$ for OS); conversely, an opposite trend was seen in node-positive patients treated with adjuvant chemotherapy (see also Table 3; $P = 0.23$ for RFS and $P = 0.16$ for OS), whereas in node-positive patients treated with hormonal therapy survival remained constant, regardless of the CEA score. These data, showing slight differences in survival depending upon stage and adjuvant treatment, are not completely comparable with the studies of Sundblad et al (1996) and Esteban et al (1994), who did not perform a separate analysis for homogeneously staged and treated patients and did not describe whether patients received any adjuvant treatment. Esteban et al (1994) reported that low CEA expression was associated with higher risk of death in the ER-negative subgroup, which is the group of breast cancer patients more frequently treated with adjuvant chemotherapy. It could be hypothesized that patients with high CEA expression could benefit more from chemotherapy than cases with low or absent CEA immunostaining. Further studies on larger series of homogeneous stage breast cancers are needed to analyse the prognostic value of CEA immunostaining, especially by analysing large subsets of homogeneously treated patients.

The present data further support the prognostic value of traditional pathological parameters, including tumour grade. Tumour grading according to Elston and Ellis (1991) is a reproducible, cost-effective and reliable tool for predicting tumour behaviour. The possibility of coupling the effects of tumour size, nodal status and tumour grade into the Nottingham prognostic index can further improve the prognostic power of classical pathological parameters (Todd et al, 1987). However, it is true that, although it is quite easy to predict the outcome of patients with the best and worst prognosis, there is still a large percentage of patients with a much more undetermined prognosis for which the search for additional biological prognosticators may be of clinical use.

Concerning the other parameters evaluated in the present study in relation to adjuvant therapy, we could confirm that ER immunohistochemical status was prognostically relevant in node-positive patients receiving hormonal therapy; conversely ER status was not prognostically useful among node-negative patients, who in the majority of cases were not treated with adjuvant therapy, and in node-positive patients receiving chemotherapy. These data are in keeping with the hypothesis that ER status as determined using immunohistochemical methods represent an important marker for predicting therapeutic response to adjuvant systemic therapy (Veronese et al, 1995; ASCO, 1996). The capability to predict therapeutic response is indeed one of the most exciting fields of clinical research: the therapeutic effect of adjuvant therapy is relatively small, and any effort should be made to use chemotherapy in a selective way to maximize the benefit to the individual patient while sparing unnecessary side-effects to patients who will not respond to therapy. ER status is actually the most reliable predictive marker, but there is indeed the need to find additional markers to couple with it to better define individual patient's characteristics. Whether CEA immunoreactivity will also prove to be an interesting marker in this respect, as suggested by Esteban et al (1994), is still to be verified (Hayes et al, 1996), but our present data do not seem very encouraging.

### ACKNOWLEDGEMENT

The authors thank Dr Hector Battifora for the generous gift of the anti-CEA antibody, for having tested a pilot series of cases and for several suggestions and comments.

### REFERENCES

ASCO (1996) Clinical practice guidelines for the use of tumour markers in breast and colorectal cancer. *J Clin Oncol* **14**: 2843–2877

Durrelman S and Simon R (1989) Flexible regression models with cubic splines. *Stat Med* **8**: 551–561

Elston CW and Ellis IQ (1991) Pathological prognostic factors in breast cancer I. The value of histological grade in breast cancer: experience from a large study with long term follow-up. *Histopathology* **19**: 403–410

Eskelinen M, Lipponen P and Syrjanen K (1992) Expression of tumour markers CA50, CEA and TPA in female breast carcinoma as related to histopathologic findings and survival. *Anticancer Res* **12**: 91–95

Esteban JM, Paxton R, Mehta P, Battifora H and Shively JE (1993) Sensitivity and specificity of Gold types 1 to 5 anticarcinoembryonic antigen monoclonal antibodies: immunohistologic characterization in colorectal cancer and normal tissues. *Hum Pathol* **24**: 322–328

Esteban JM, Felder B, Ahn C, Simpson JF, Battifora H and Shively JE (1994) Prognostic relevance of carcinoembryonic antigen and estrogen receptor status in breast cancer patients [see comments]. *Cancer* **74**: 1575–1583

Gion M, Mione R, Dittadi R, Fasan S, Pallini A and Bruscagnin G (1986) Carcinoembryonic antigen, ferritin, TPA in serum and tissue: relationship with the receptor content in breast carcinoma. *Cancer* **57**: 917–922

Harrell FE, Califf RM, Pryor DB, Kerny LL and Rosati RA (1982) Evaluating the yeld of medical tests. *J Am Med Assoc* **247**: 2543–2548

Hayes DF, Bast RC, Desch CE, Fritsche H, Kemeny NE, Jessup JM, Locker GY, Macdonald JS, Mennel RG, Norton L, Ravdin P, Taube S and Winn RJ (1996) Tumor marker utility grading system: A framework to evaluate clinical utility of tumor markers. *J Natl Cancer Inst* **88**: 1456–1466

Kuhajda FP, Offutt LE and Mendelshon G (1983) The distribution of carcinoembryonic antigen in breast carcinoma. Diagnostic and prognostic implications. *Cancer* **52**: 1257–1264

Levesque MA, Diamandis EP, Yu H and Sutherland DJ (1994) Quantitative analysis of mutant p53 protein in breast tumor cytosols and study of its association with

© Cancer Research Campaign 1998 

other biochemical prognostic indicators in breast cancer. *Breast Cancer Res Treat* **30**: 179–195

Mansour EG, Hastert M, Park CH, Koehler KA and Petrelli M (1983) Tissue and plasma carcinoembryonic antigen in early breast cancer. A prognostic factor. *Cancer* **51**: 1243–1248

Molina R, Zanon G, Filella X, Moreno F, Jo J, Daniels M, Latre ML, Gimenez N, Pahisa J and Velasco M (1995) Use of serial carcinoembryonic antigen and CA 15.3 assays in detecting relapses in breast cancer patients. *Breast Cancer Res Treat* **36**: 41–48

Neumeier M, Shively L, Chen FS, Gaida FJ, Ilgen C, Paxton RJ, Shively JE and Riggs AD (1990) Cloning for the genes for T84.66, an antibody that has a high specificity and affinity for carcinoembryonic antigen, and expression of chimeric human-mouse T84.66 genes in myeloma and Chinese hamster ovary cells. *Cancer Res* **50**: 2128–2134

Sundblad AS, Pellicer EM and Ricci L (1996) Carcinoembrionic antigen expression in stages I and II breast cancer: its relationship with clinicopathologic factors. *Hum Pathol* **27**: 297–301

Todd DW, Elston CW, Ellis IO, Hilton RW, Blamey RW and Haybittle JL (1987) Confirmation of a prognostic index in primary breast cancer. *Br J Cancer* **56**: 489–492

Veronese S, Barbareschi M, Morelli L, Aldovini D, Mauri FA, Caffo O, Gambacorta M and Dalla Palma P (1995) Predictive value of ER1D5 antibody immunostaining in breast cancer. *Appl Immunohistochem* **3**: 85–90

Walker RA (1980) Demonstration of carcinoembryonic antigen in human breast carcinomas by the immunoperoxidase technique. *J Clin Pathol* **33**: 356–360

*© Cancer Research Campaign 1998*